# Unsupervised, frequent and remote: A novel platform for personalised digital phenotyping of long-term memory in humans

**DOI:** 10.1371/journal.pone.0284220

**Published:** 2023-04-26

**Authors:** Marius Bauza, Marino Krstulovic, Julija Krupic

**Affiliations:** 1 Sainsbury Wellcome Centre, University College London, London, United Kingdom; 2 Department of Physiology, Development and Neuroscience, University of Cambridge, Cambridge, United Kingdom; University of Plymouth, UNITED KINGDOM

## Abstract

Long-term memory tests are commonly used to facilitate the diagnosis of hippocampal-related neurological disorders such as Alzheimer’s disease due to their relatively high specificity and sensitivity to damage to the medial temporal lobes compared to standard commonly used clinical tests. Pathological changes in Alzheimer’s disease start years before the formal diagnosis is made, partially due to testing too late. This proof-of-concept exploratory study aimed to assess the feasibility of introducing an unsupervised digital platform for continuous testing of long-term memory over long periods outside the laboratory environment. To address this challenge, we developed a novel digital platform, hAge (‘healthy Age’), which integrates double spatial alternation, image recognition and visuospatial tasks for frequent remote unsupervised assessment of spatial and non-spatial long-term memory carried out continuously over eight week period. To demonstrate the feasibility of our approach, we tested whether we could achieve sufficient levels of adherence and whether the performance on hAge tasks is comparable to the performance observed in the analogous standard tests measured in the controlled laboratory environments.191 healthy adults (67% females, 18-81 years old) participated in the study. We report an estimated 42.4% adherence level with minimal inclusion criteria. In line with findings using standard laboratory tests, we showed that performance on the spatial alternation task negatively correlated with inter-trial periods and the performance levels on image recognition and visuospatial tasks could be controlled by varying image similarity. Importantly, we demonstrated that frequent engagement with the double spatial alternation task leads to a strong practice effect, previously identified as a potential measure of cognitive decline in MCI patients. Finally, we discuss how lifestyle and motivation confounds may present a serious challenge for cognitive assessment in real-world uncontrolled environments.

## Introduction

Neuropsychological tests provide a powerful tool for facilitating the diagnosis of Alzheimer’s disease (AD) and other forms of dementia [1–3]. At the early stage of AD, the Tau pathology is almost exclusively confined to the medial temporal lobe regions (MTL) [4], which is crucial for episodic [5–7], verbal [8], and visuospatial memory [6, 9], image recognition [6, 10] and navigation [11, 12]. Hence, neuropsychological tests targeting these functions were shown to be especially suitable for early AD detection and its differentiation from other neurological conditions such as depression [1, 2]. Such tests are normally applied every 8–24 months in research laboratories and clinics by a trained specialist to monitor any changes in the condition [1, 3]. As a result, the implementation of such tests is limited in scale, frequency, and duration, which was especially exacerbated during the COVID-19 pandemic [13]. This creates an urgent need to introduce new approaches, implementable at scale and frequency. Here, we present a novel digital platform called hAge (‘healthy Age’) for a fully unsupervised remote and frequent long-term assessment of spatial long-term memory in real-world ethologically-relevant environments (i.e. Participants’ homes, workplaces, etc.). hAge platform is based on standard MTL-specific tests executed in a game-like form, which involves frequent few-seconds-long interactions throughout the day. hAge includes image recognition, visuospatial and spatial alternation tasks, designed to probe long-term memory and known to be highly sensitive to damage in the MTL [6, 9, 14] and prefrontal areas [9, 15], both compromised in the early and middle stages of AD [4]. It has been suggested that image recognition may be sensitive to either hippocampal formation or extra-hippocampal MTL regions (perirhinal, lateral entorhinal and parahippocampal cortices), depending on the strategy an individual uses to solve the task. The image recognition task can be solved using a familiarity strategy (i.e. the image was seen before vs novel) or a recall. The use of a cognitively less demanding familiarity strategy has been proposed to rely on extra-hippocampal MTL (perirhinal and entorhinal regions) [16, 17], while the use of the cognitively more demanding recall strategy is thought to depend on the hippocampus [10, 17], but see [18]. The visuospatial memory task has been shown to depend on both the hippocampus and the prefrontal areas [9]: since both images are familiar, the familiarity strategy cannot be used to solve the visuospatial (i.e. image-in-place) memory task. Notably, the performance on both image recognition and visuospatial memory tasks is expected to be impaired in presymptomatic AD patients [16, 17] due to the impairments in perirhinal and entorhinal regions at the early stages of the disease (Braak stages I and II) [4], whereas impairment in spatial alternation task is likely to be apparent only at the later stages of the disease (Braak stages III and IV) when the hippocampal damage is observed [4]. Applying these tasks together could help determining the disease stage and the affected regions.

To demonstrate the feasibility of our approach, we tested whether we could achieve sufficient levels of adherence and whether the performance on hAge tasks is comparable to the performance observed in the analogous standard tests measured in the controlled laboratory environments. Based on previous studies in humans and rodents [9, 19], we expected performance on the spatial alternation task to decrease with longer time intervals between trials. Furthermore, we predicted that performance could be modulated by adjusting the difficulty of the tasks, e.g., selecting more similar images or introducing more complex alternation rules. We also expected performance improvements with repeated exposure, known as practice effects [20–22]. Finally, we expected to find that performance may negatively correlate with age [23, 24]. All of these measures were previously used to assess cognitive decline and the risk of developing AD in MCI patients [20–22, 25]. In particular, it has been shown that patients with mild AD and amnesic MCI perform significantly worse on image recognition and visuospatial tasks than controls; and that the difference in performance increases with the inter-trial interval [25]. Moreover, it has been shown that repeated engagement in various cognitive memory tasks (e.g. visuospatial Memory Test, Hopkins Verbal Learning Test etc.) results in improved long-term performance in controls but hardly any improvement in amnesic MCI patients [20–22].

## Methods

### Participants

Nulla mi mi, Fig venenatis sed ipsum varius, volutpat euismod diam. This study has been approved by the University of Cambridge Psychology Research Ethics Committee (PRE.2020.053). Written informed consent was obtained from all study Participants, and the data were analysed anonymously. Participants were recruited via the Cambridge Psychology Research sign-up system and the Join Dementia Research database. Volunteers were included in the study if they declared that they met the following criteria: (1) above 18 years old; (2) had no visual or hearing impairments; (3) had no learning disabilities or dementia; (4) had no history of neurological and psychiatric disorders; (5) had access to a Windows PC. Engagement rules were provided in the Participant Information Sheet and the Participant Consent Form and clarified via email, phone call or Zoom session upon request. Participants were explicitly asked to participate in the study for eight weeks and were offered a small £10/week payment if they engaged with the program on average >15 times/day, paid at the end of each week via bank transfer or cheque. After eight weeks, Participants were notified that their participation period has ended but they could continue using the App.

The program was run in two separate 8-week phases (Phase 1 and Phase 2). The participation data were stored on local user computers. The data was encrypted and uploaded to a secure University of Cambridge website several times a day. After the study was concluded, data was anonymised by deleting the table linking the Participants’ identities and aliases.

### hAge tasks and design

**Fig 1:**
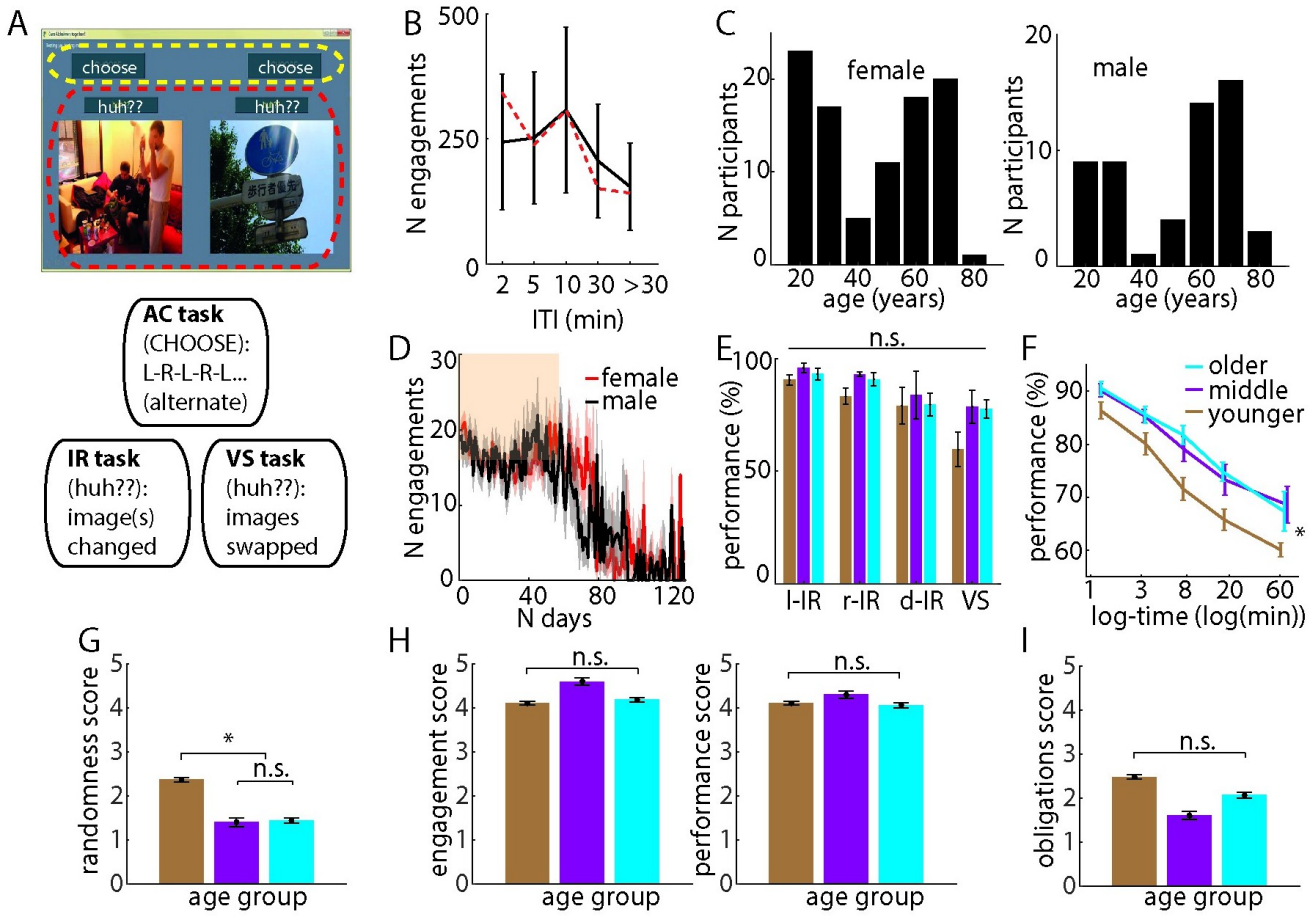
Phase 1 testing of hAge feasibility. A: Phase 1 hAge task design. The program consists of three types of tasks: 1) spatial alternation (SA), during which Participants clicked one of the [choose] buttons (yellow inset), alternating between left (L) and right (R) on every engagement; 2) image recognition (IR) and 3) visuospatial (VS) task (red inset): Participants clicked [huh??] button above the image that was perceived to have changed (left or right IR, l-IR and r-IR, respectively). Both left and right [huh??] buttons were clicked when both images changed (d-IR) or swapped sides (VS). B: Most of the time, the Participants engaged with the program shortly after it became active. Black solid line indicates the actual distribution of inter-trial intervals (ITIs±s.d.). Red dashed line indicates the hard-coded normalized ITI distribution at which the hAge window became active. C: Female (left) and male (right) distribution by age before applying exclusion criteria. D: Daily engagement levels throughout the study. Zone of rewarded participation (8 weeks) is shown in orange. Red and black mark female and male engagement levels, respectively. E: All age groups showed high performance on IR and VS tasks. F: Performance on the SA task was negatively correlated with the inter-trial period in all age groups. On average, the performance in younger group was significantly poorer (indicated by an asterisk) compared to the older groups. G: The average self-reported randomness score shows how often Participants clicked a random button to complete their engagement with the program. 1 to 5=’never’ to ‘very often’. H: The average self-reported engagement score (left) shows how motivated the Participants were to achieve the daily required engagement quota, and the average performance score shows how motivated the Participants were to do well when providing their answers. 1 to 5 = ‘not motivated’ to ‘extremely motivated’. I: The average self-reported obligations score shows how overwhelmed the Participants were with other professional and/or social/personal obligations. 1 to 5 = ‘not at all’ to ‘extremely overwhelming’. Different colours represent different age groups and are maintained throughout the figure.

The hAge program was designed to simultaneously test three different types of medial temporal lobe (MTL) dependent tasks [14] over a long period in a completely unsupervised way. The first set of tasks was variants of a spatial alternation task (Fig 1A, a yellow inset). The second set involved either image recognition or image-in-place (also known as visuospatial) recognition tasks (Fig 1A, a red inset). Both sets were run simultaneously on the Participants’ computers. The hAge program (research.pdn.cam.ac.uk/hkage) was custom-written in Python for Windows OS.

The hAge interface comprised two images and two pairs of buttons, labelled **[choose]** and **[huh??]**, displayed in the same grey window presented at random time intervals (Fig 1A). The time interval between the window appearances (inter-trial interval) varied from ∼25 seconds to ∼3 hours (Fig 1B). The program window was invisible at all other times to prevent any ‘rehearsal’. A gentle repetitive sound was played to signal that the program was activated. The program window remained active until the engagement was completed (i.e. Participants indicated their choices). The Participant could also ignore the prompting and re-engage at their own time. During the engagement period, the Participants were presented with both the spatial alternation and the image recognition tasks within the same window (Fig 1A, yellow and red insets, respectively). In Phase 1 (see below for details), to make a correct choice on the spatial alternation, Participants had to alternate their responses between the trials by clicking the left or right **[choose]** buttons. In Phase 2 (see below for details), the Participant had to alternate on every second choice. The concurrently presented image recognition task required Participants to click one or both of the **[huh??]** buttons if they thought the image beneath the button had changed since the last engagement. If both images changed or swapped places, the Participants were required to click both **[huh??]** buttons. Participants were given instructions on how to conduct the tasks before commencing their engagements.

Previously, it has been shown that subjects with MTL lesions were impaired even on ‘classic’ working memory tasks (e.g. remembering digit or/and letter sequences [26, 27]) compared to controls whenever (1) there was interference between the tasks, (2) subjects were operating at their working memory capacity, or (3) there was a long maintenance period between the tasks (see review by Jeneson and Squire [14]). These results suggest that declarative long-term memory is required to retrieve information, which dropped out from the focus of attention due to distractors or long (> several seconds) inter-trial intervals [14, 28]. Distractions and long inter-trial periods are the key properties of our testing platform.

### Two hAge testing phases

The hAge program was tested in two phases (Phase 1 and Phase 2). We used Phase 1 as a proof-of-principle pilot experiment to test the feasibility of our approach. To this end, we implemented the simplest versions of the tasks: images in the image recognition and visuospatial tasks were drawn from completely different categories (e.g. people vs highways, etc.), and the rule of the special alternation task was to alternate between consecutive choices in order to make a correct choice. Images were alternated on average once a day. In Phase 1, we used a disbalanced ratio of single image recognition tasks (N.B. we planned to use this to check whether the side bias would affect the type of changes the Participants anticipated; we found that it did, but this is out of scope of the current work): 53% on one side (e.g. an image on the left was changed, l-IR) and 13% on the opposite side (e.g. an image on the right was changed, r-IR; for each person, it was randomly decided which side bias was introduced, and the same bias was used throughout; the choice of a bias was balanced between Participants). 13% of instances corresponded to both images being changed (d-IR); and 20% of instances corresponded to visuospatial task (VS) where image identities remained the same, but their locations were swapped.

In Phase 2, we made some alterations to the program code to fix technical glitches reported by the Participants. Based on their feedback, we also altered the program’s activation sound (see below). Finally, all tasks were made substantially harder to provide a greater spread of the performance scores. Namely, simultaneously displayed images were drawn from the same category (planes, dogs, highways or buildings), and the tasks were run more frequently compared to Phase 1: on average, 5 image changes per day. 40% and 25% of instances involved a single image change (as in Phase 1, we introduced the bias towards one of the sides, and the bias choice was balanced between the Participants); 17% of cases represented instances where both images were changed (d-IR); and 17% of instances corresponded to visuospatial task where image identities remained the same, but their locations were swapped. In Phase 2, a single alternation task was changed to a double spatial alternation task, which required the Participant to alternate sides on every second choice. In both Phases, a Participant was immediately provided with feedback on whether their choice was correct on alternation tasks but not on image recognition or visuospatial tasks.

### Inclusion criteria

To provide a reliable longitudinal assessment of long-term spatial memory, we only included data from Participants who had engaged with the spatial alternation task for at least 6 out of 8 weeks with a daily engagement of >10/day. The duration and daily engagement were decided prior to the data collection. The image recognition or visuospatial tasks were included only when the former criterion was met and when the total number of engagements in the image recognition or visuospatial tasks was >10 times in total (for each of these tasks). To assess learning (practice) effects on the image recognition tasks, the performances were split into two equal blocks (early vs late) and were included in the final analysis if there were at least ten measurements on each task per block. During Phase 2 frequency of image changes was increased from ∼1 change/day (Phase 1) to ∼5 changes/day (Phase 2) to assess learning effects.

We also excluded a small number of Participants (n = 17, S1 Table in S1 Appendix) who showed abnormally high (average performance >90% across all ITIs) or unusually low-performance levels (<60%) under the assumption that they likely misunderstood the task by using external reminders (high performers, n = 7) or choosing mostly randomly (low performers, n = 10). The criteria for outliers were chosen a priori before commencing the study. It must be noted that the main results do not change if we do not exclude these Outliers. The final number of Participants included in the final analysis is shown in S2 Table in S1 Appendix and in the participation flowchart diagram (S1 Fig in S1 Appendix).

### Spatial alternation and double spatial alternation tasks

The performance on the alternation tasks was evaluated at five different inter-trial intervals (ITI): (1) <2min; (2) 2–5 min; (3) 5–10 min; (4) 10–30 min, and (5) 30min-5.5 hours (the maximum ITI set by the program was 3 hours; however, in some cases, Participants engaged with the App at some later times, Fig 1B) to ensure adequate sampling for each interval (>100 samples per interval). The median of all values within a respective bin is shown as the position of that bin in plots. The ITIs are displayed as log(min) to facilitate data visualisation. The performance on the double alternation task was divided into win-shift (Participants had to alternate a previously chosen side to make a correct choice) and win-stay task (Participants had to hold on to a previously chosen side to make a correct choice).

### Statistical analysis

The data was analysed using MATLAB (The MathWorks Inc.). All statistical tests are stated in the main text and Supplementary Methods. The performance dependence on ITIs was assessed using repeated measures ANOVA. The paired-sample t-test was used to estimate if there was a significant difference in performance on the single and double alternation tasks and between the win-stay and win-shift components of the double alternation task. The average performance across all ITIs was used for comparisons. One sample t-test was used to estimate if there was a significant difference between IR and VS tasks in different Phases. One-way ANOVA with Bonferroni correction was used to estimate the effect significance of different age groups. Single and double image recognition tasks were combined for statistical analysis.

The Kruskal-Wallis H test was used to compare the questionnaire results between different age groups.

The chance level for image recognition and visuospatial tasks was calculated by assuming that upon reactivation of the program window, a Participant would randomly select that one or both images changed, with an equal probability between all four visual tasks.

### Correlation analysis

Spearman’s rank correlation coefficient was used to estimate the change in performance on double spatial alternation task over days.

### Feedback collection

At the end of the eight weeks, Participants were informed that their participation was completed and they would no longer receive any payment, although they could continue using hAge if they wished to, and the collected data would be used in the study. We also asked Participants to complete a short feedback form and a demographics questionnaire (S2 Fig in S1 Appendix). Phase 2 Participants, who passed the inclusion criteria, were additionally asked to complete a short follow-up questionnaire regarding their lifestyle and motivation (here, by motivation, we mean willingness to adhere to the task requirements) while they used the hAge program (S1 File in S1 Appendix) and were invited to participate in the Focus Group discussion (S2 File in S1 Appendix) to help better understand their experience of performing the tasks and the strategies they used to solve them. The questionnaire consisted of 5 questions which were on a 5-point Likert scale. Out of the 72 people that were emailed, 45 (62.5%) responded to the questionnaire. Of the 45 respondents, 16 were from the older, 10 were from the middle and 19 were from the younger age groups. 10 Participants were selected for the follow-up Focus groups. Each Focus Group comprised 3–4 Participants, and two researchers involved in the study, and the meeting lasted ∼1 hour. Altogether, we conducted 3 Focus group meetings.

## Results

### Phase 1: Demonstrating the feasibility of hAge for longitudinal, remote and unsupervised assessment of long-term memory

In total, 151 healthy adults (18–81 years old; mean±s.d.: 48±20 years old; 63% females, Fig 1C, S2 Fig, S3 Table in S1 Appendix) took part in Phase 1, of whom 22% (67% females, S3 Fig in S1 Appendix) passed the inclusion criteria (S1, S2 Figs in S1 Appendix; engaged with the App for at least six weeks, see Methods) and were used for subsequent analysis. It must be noted that the majority of Participants were excluded due to an ‘insufficient’ number of days they engaged with the program (S4 Fig in S1 Appendix; less than 42 out of 56 days; with the majority of Participants engaging for at least ∼30 days) and not due to the low number of daily engagements. The age distribution was comparable between these two populations. On the other hand, we also found that many Participants continued to play beyond the requested 8-week period (Fig 1D, S4 Fig in S1 Appendix) at high daily engagement levels, with many reporting that they liked using the program and wished to continue playing (S4 Table in S1 Appendix), supporting the feasibility of such active frequent unsupervised testing.

To assess whether performance on various memory tasks varied as a function of age, Participants were divided into older (>65 years old), middle (50–60 years old) and younger (<50 years old) age groups. All age groups showed high performance on image recognition and visuospatial tasks with no significant difference between the groups (Fig 1E; average combined IR and VS tasks: F(2,30)=0.44, P = 0.65, one-way ANOVA test). The majority of errors on the visuospatial task resulted from missing the change.

In line with our initial hypothesis, we also found that performance on the spatial alternation task rapidly decreased with longer ITIs (Fig 1F; F(4,124)=43.67, P = 6.4 × 10^−13^ with Greenhouse-Geisser correction, repeated measure ANOVA). However, unexpectedly, the average combined performance was lowest in the younger group, with no differences between the middle and older groups (Fig 1F, mean±s.m.e.: 72.8 ± 1.5%, 79.3±2.5%; 80.0±1.9% in younger, middle and older age groups respectively; P = 0.009, F(2,30)=5.53, one-way ANOVA) even though the performance on analogous clinic-based memory tests typically negatively correlates with age [29]. Based on previous studies [30, 31] and the results from the follow-up Questionnaire (S1 File in S1 Appendix), higher absolute performance of the middle and older groups may be attributed to differences in commitment levels, with older and middle groups likely dedicating more cognitive effort in an attempt to correctly complete the task. Namely, when the Participants were asked if they often made a random choice to end the engagement and return to their previous activities, the younger group did this significantly more often compared to older and middle age groups (Fig 1G, randomness score mean±s.m.e.: 2.4±0.3, 1.4±0.2; 1.4±0.2 in younger, middle and older age groups respectively; P = 0.01, X2 (2, 42) = 9.2, Kruskal-Wallis H test). Interestingly, the groups’ subjective perception of commitment and motivation was not significantly different (Fig 1H). In addition, the younger group tended to have higher levels of perceived distraction, possibly due to more active lifestyles, although the difference was not significant (Fig 1I, obligations’ score mean±s.m.e.: 2.5±0.2, 1.6±0.3; 2.1±0.2 in younger, middle and older age groups respectively; P = 0.099, X2(2, 42)=4.63, Kruskal-Wallis H test). The assumption of lower commitment levels in a younger group was further corroborated by a steep decline in average performance of a younger group on the spatial alternation task, which happened after the required 8 weeks after which participants were notified about the end of their participation and weekly participation payments were discontinued; and which was not observed in the older and middle groups (S5 Fig in S1 Appendix). On the other hand, the observed decline in younger group could also be explained by the higher cognitive load as most of the Participants from the younger group were also using their computers for work.

### Phase 2: Evaluating performance on more challenging tasks

**Fig 2:**
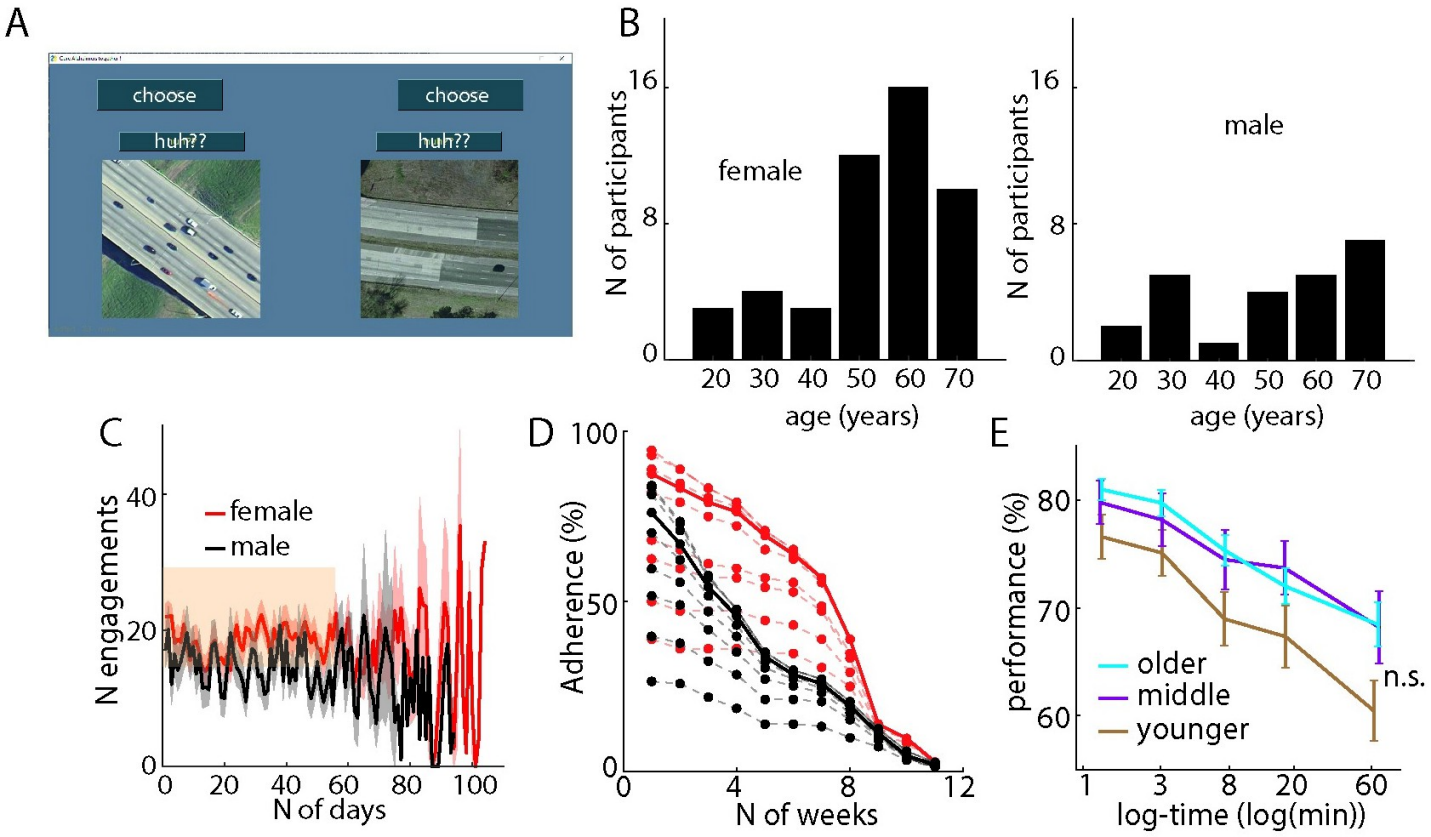
Performance on Phase 2 hAge program. A: Phase 2 hAge was significantly more challenging compared to Phase 1. The SA task was changed to a double spatial alternation (dSA) task where Participants had to alternate on every second choice to pick the correct side (LLRRLLRR). Simultaneously presented images on IR and VS tasks were drawn from the same categories. B: Female (left) and male (right) distribution by age. C: Daily engagement levels throughout the study. Zone of rewarded participation is shown in orange. D: The adherence levels at different durations and daily participation in Phase 1 (black) and Phase 2 (red) programs. The x-axis corresponds to the number of weeks of engagement. Solid lines show the minimum daily engagement level equal to 10 used in the analysis. Dashed lines correspond to minimum daily engagement levels set to 4, 6, 8 (above solid lines) and 12, 14, 16, 18, and 20 (below solid lines). As expected, the adherence levels fall with the increased daily engagement and the total required duration of the engagement. E: As in the Phase 1 SA task, the performance on the dSA task was negatively correlated with the inter-trial period in all age groups. There was no significant difference in performance between different age groups.

The second, more challenging version (Phase 2) of the hAge program with modified SA, VS and IR tasks increased the cognitive demand required to solve them. Based on findings from analogous standard tests, we hypothesised that the Participants would show reduced performance on all tasks. This is important because the high average performance observed during Phase 1 may lead to a ceiling effect and limit the usability of our approach. Instead of a simple SA task, we implemented a double spatial alternation (dSA) rule (see Methods). Furthermore, co-displayed images in IR and VS tasks were drawn from the same image category, making them significantly more similar and hence more challenging to spot the change (Fig 2A). In total, 72 adults (19–73 years old; mean±s.d.: 53±15 years-old; 67% females) participated in the Phase 2 release (Fig 2B), of whom 54% passed the inclusion criteria (22–73 years old, 74% females; Fig 2C and 2D). 44% (32/72) also participated in Phase 1 to allow direct comparison. Importantly, of the 40 new Participants taking part in Phase 2, 53% (21/40) passed the inclusion criteria suggesting that modifications introduced in Phase 2 (fixing technical glitches, changing the activation sounds and modifying the cognitive tasks) were able to significantly improve the adherence levels compared to Phase 1. However, it should be noted that we cannot rule out that the improved adherence may be affected by the inclusion of more motivated Participants from Phase 1 who changed their IDs and were not identified as such. Furthermore, the included Phase 1 and Phase 2 Participants had some differences in the age distribution (S3 Fig in S1 Appendix), which may have also introduced some differences in the adherence levels.

**Fig 3:**
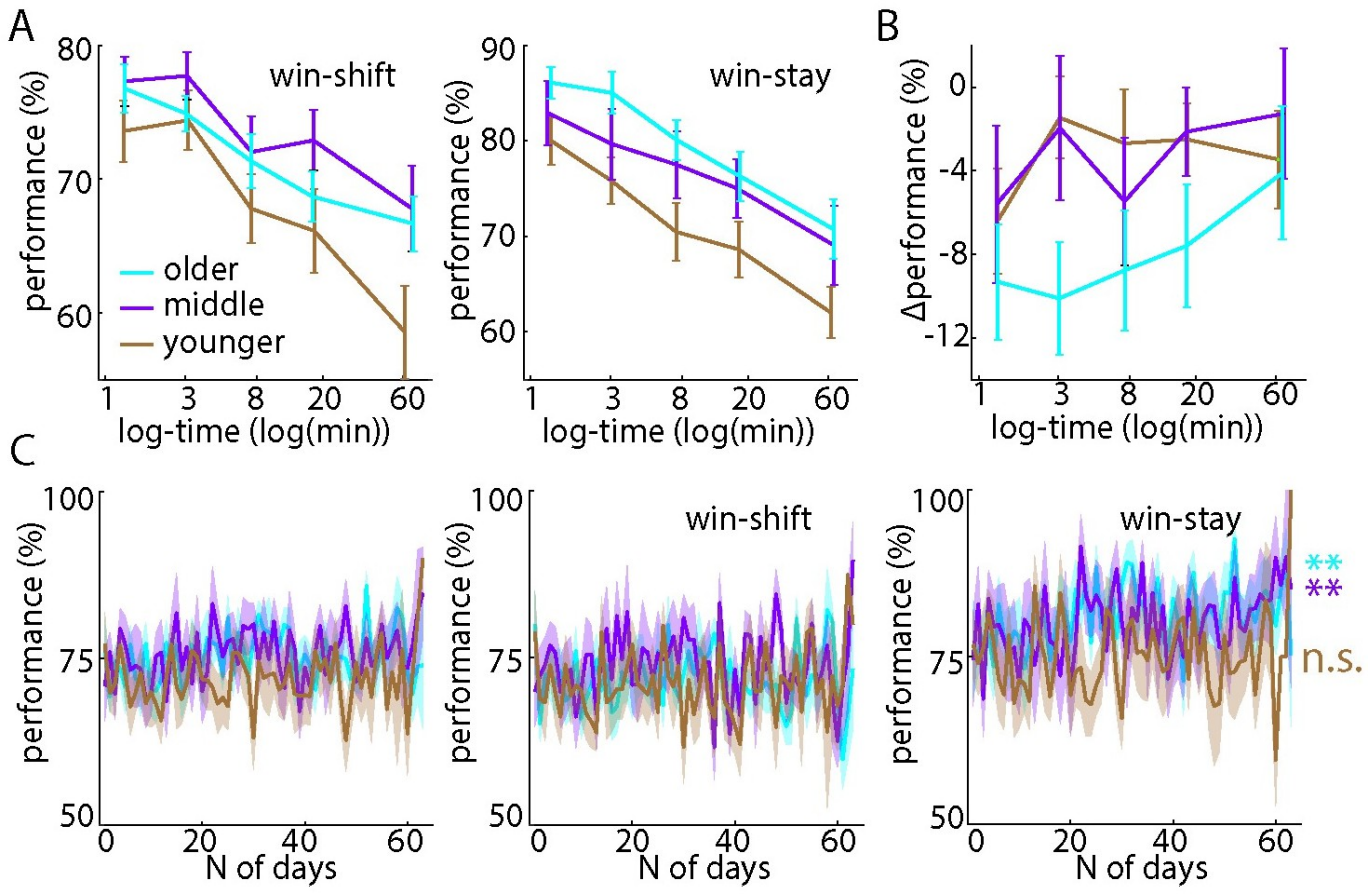
Different performances on win-shift and win-stay dSA tasks. A: All age groups showed different performances on win-shift (alternate the choice) vs win-stay (keep the same choice) dSA tasks. B: There was no difference in relative performance (difference between performance on win-shift and win-stay tasks) between different age groups. C: Overall performance (left) did not change with experience in any age group. However, while the performance on the win-shift task requiring remembering alternate choices did not improve with experience, the performance on the win-stay task (i.e. remembering to hold on to the same choice) kept on significantly improving in the older and middle groups but not in the younger group.

As expected, the Participants who took part in Phase 1 and Phase 2 performed significantly worse on dSA compared to the SA task (mean difference ± s.m.e.: 9.7±0.2%; P = 0.017, t=-2.53, df = 27 paired-sample t-test), likely due to increased memory load requiring to remember the last choice and the order within the choice sequence. Similar to Phase 1, the performance on the dSA task declined with increasing ITIs (Fig 2E and 2F(4, 148)=13.76, P = 1.05 × 10^−6^ with Greenhouse-Geisser correction, repeated measure ANOVA). However, unlike in Phase 2, there was no longer a significant difference in average performance between different age groups (Fig 2E; P = 0.11, F(2, 36)=2.38, one-way ANOVA). Interestingly, performance on the dSA task varied depending on the type of choice Participants made. Namely, in one case, the correct choice was the opposite of the previously selected one, requiring to alternate (win-shift), whereas in another case, the Participant had to repeat the previous choice (win-stay). The average performance on the win-shift task was significantly lower compared to the win-stay task (Fig 3A, mean difference±s.m.e.: 7.6±1.3%, P = 4.1 × 10^−4^, t = -3.87, df = 38, paired sample t-test), suggesting that Participants found it more difficult to remember to alternate compared to holding on to the same side. There was no significant difference in performance on win-shift and win-stay tasks (the relative performance) between the groups (Fig 3B, mean±s.m.e.: P = 0.22, F(2,36)=1.58, one-way ANOVA).

**Fig 4:**
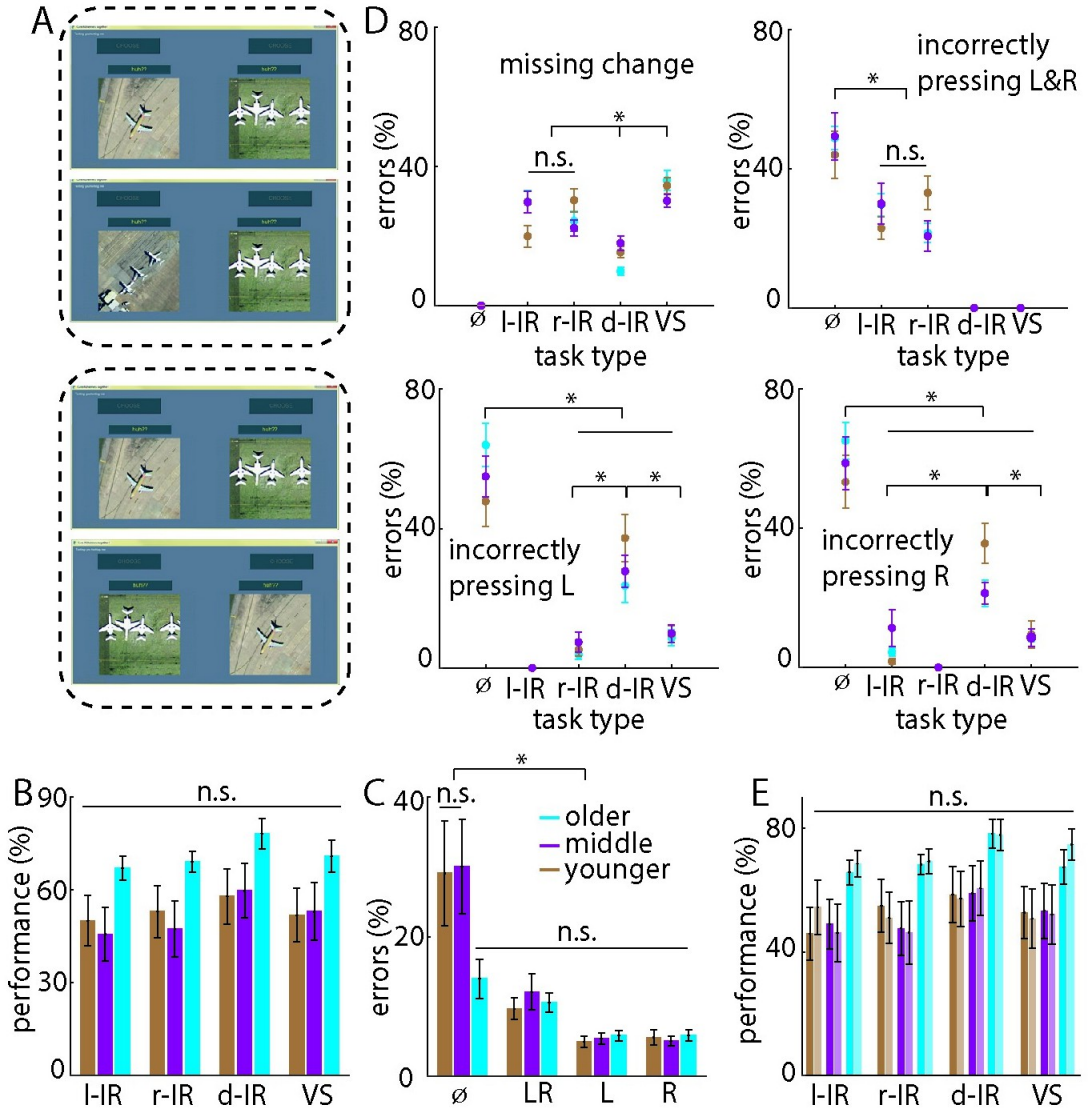
Performance on Phase 2 IR and VS tasks. A: An example of the left l-IR (top inset) and VS (bottom inset) tasks. All of the images were drawn from the same ‘planes’ category. B: Performance on Phase 2 IR and VS tasks was lower than Phase 1. C: The type of errors on IR and VS tasks for each age group. ‘Missing change’ errors occurred significantly more often than other errors in younger and middle age groups (P<0.01 after Bonferroni correction). There was no significant difference between different types of errors in the older group. D: Breakdown of choices associated with different types of errors (specified in legend). Task types are shown on the x-axis. fucking-diameter-sign denotes no image change taking place. All age groups missed the change in VS task significantly more often compared to other image recognition tasks (P<0.012 after Bonferroni correction), while the missing change occurred significantly more seldom when both images changed (P<10^−5^ after Bonferroni correction, top left). The Participants tended to significantly overreact to no change (fucking-diameter-sign) by incorrectly pressing left (L), right (R) or both (LR) buttons (P<10^−5^ after Bonferroni correction, top right). E: The performance on different IR and VS tasks did not noticeably improve with experience: the first half of total engagements are shown in the dark, and the latter half in light colours for each age group.

We next investigated whether our longitudinal assessment allowed us to detect any practice effects previously used to evaluate cognitive decline in MCI patients [20–22]. To address this question, we looked at whether there were any changes in win-shift and win-stay task performance over time. Indeed, the performance of older and the middle groups on the win-stay task improved significantly with experience (Fig 3C, *ρ*=0.40, P = 0.0013; and *ρ*=0.36, P = 0.004, respectively; Spearman’s Rank-Order Correlation) while the performance on the win-shift task on average remained similar over time (*ρ* =-0.03, P = 0.80 and *ρ*=0.002, P = 0.99, respectively; Spearman’s Rank-Order Correlation). Notably, the performance did not improve with experience in the younger group on either win-stay or win-shift tasks (*ρ*=0.089, P = 0.49 and *ρ* =0.14, P = 0.27, respectively; Spearman’s Rank-Order Correlation). As expected, the overall performance on image recognition and visuospatial tasks (Fig 4A) was significantly lower in Phase 2 compared to Phase 1 in all of the Participants who took part in both Phases of the study (Fig 4B; mean difference ± s.m.e.: 49.0±5.4%, P = 5.4 × 10^−8^, t=-7.5, df = 26 one-sample t-test). The new task provided a wide spread of scores, avoiding both ceiling (100% performance) and floor (chance level ∼5%, see Methods) effects. As in Phase 1, there was no significant difference in performance between different age groups (combined average performance on IR and VS tasks mean±s.m.e.: 71.2±1.2%, 51.4±1.1% and 53.1±1.1% in the older, middle and younger groups, respectively P = 0.091, F(2, 35)=2.57). In general, given relatively high image similarity, most errors for all age groups occurred when the Participants failed to notice any change, which was more pronounced in the middle and younger groups, while the other types of errors remained similar between the groups (Fig 4C). The majority of such false negatives were made during VS task, while the least occurred on the d-IR task, reflecting the different difficulty levels of these tasks (Fig 4D). There was no overall noticeable improvement in VS or IR tasks in any age group over time (Fig 4E).

### Defining minimal inclusion criteria

**Fig 5:**
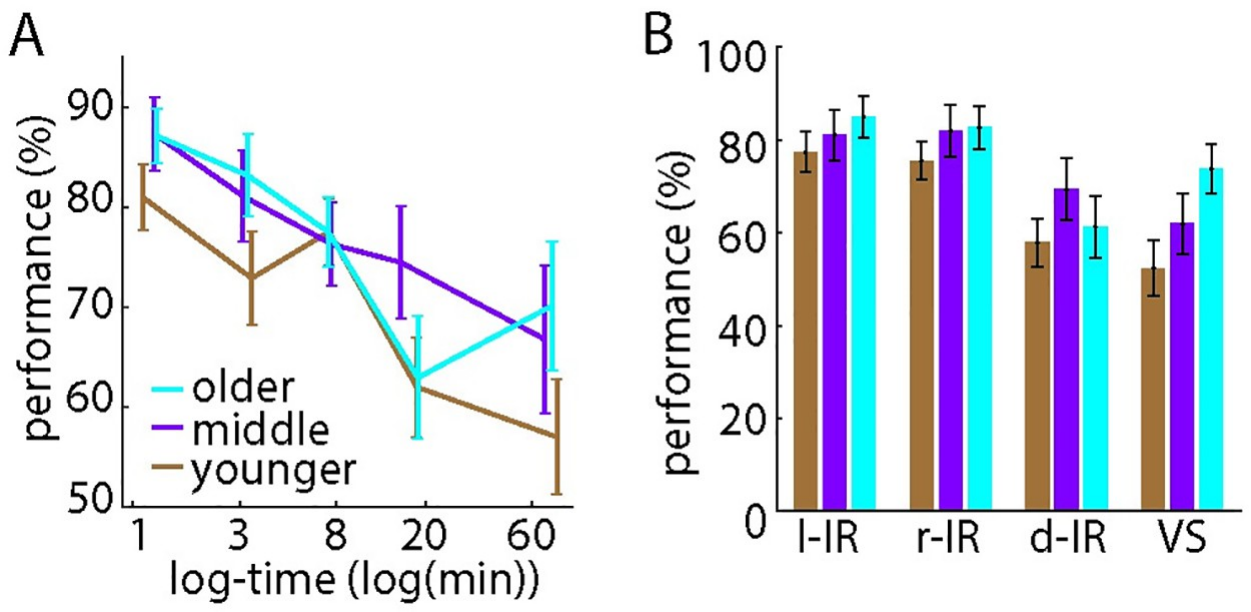
Performance on Phase 1 with adjusted minimal inclusion criteria. A: Performance on the first day of engagements in all Phase 1 Participants who used the program at least twice on that day (n = 144). The performance was negatively correlated with the inter-trial period in all age groups. There was no significant difference in performance between different age groups. B: Performance on IR and VS tasks using up to four engagements with each type of task.

In the analysis above, we used only Participants who passed our a priori defined inclusion criteria, which, due to the exploratory nature of the current study, was set out as rather conservative (at least 42 days of participation with daily engagement of >10 engagements/day) to ensure adequate statistical power. Next, we aimed to establish a set of minimal requirements for the number of engagement days and daily engagement levels, which would not qualitatively change the main conclusions of the current study. To address this question, we analysed how the performance on the AC task changed as a function of ITI on the first day of engagement in the Phase 1 dataset (144/151 Participants; Participants had to engage in the AC task at least twice on the first day to be included: Three Participants engaged less than two times on the first day of participation from the younger, three from the older and one from the middle group). We found that one day of engagement was sufficient to demonstrate that the performance on the AC task dropped significantly with longer ITIs (Fig 5a, F(4, 328)=3.24, P = 0.0196 with Greenhouse-Geisser correction, repeated measures ANOVA) with no difference in average performance across age groups (F(2, 139)=1.3, P = 0.27 one-way ANOVA). The average daily engagement level of included Participants was equal to (mean± s.d.) 19±12. Using these newly adjusted inclusion criteria (>19 engagements on the first day), the expected adherence level is 42.4% (64/151).

We next aimed to establish the minimum number of days required to reliably detect the improvement in performance with practice. Using the Phase 2 data set (39/72; with the initial a priori established criteria), we found that in the older age group, the practice effect becomes significant in approximately two weeks (Fig 3C; *ρ*=0.63, P = 0.019; the average number of daily engagement (mean±s.d.): 21±13); however, it takes approximately a month (30 days) for the practice effect to become significant in the middle age group (*ρ*=0.45, P = 0.013; the average number of daily engagement in this group (mean±s.d.): 13±7). Note that the average daily engagement was lower in the middle age group compared to the older group during the first two weeks, which may have resulted in taking longer for the practice effect to become apparent.

Finally, we investigated whether a minimum sample of four in each image recognition and the visuospatial test would be sufficient to show similar performance as in Fig 1E obtained using larger sample sizes. Indeed, we found qualitatively similar results (Fig 5b) using the entire Phase 1 data set (151 Participants with up to four engagements per Participant in each type of task).

## Discussion

Here we described a novel digital game-like platform for remote longitudinal unsupervised and frequent assessment of long-term memory known to rely on the hippocampal-trans-entorhinal circuitry in humans and other mammals [6, 8–10, 14, 32–35]. Based on Phase 1 results, we report a 42.4% Participant adherence level, which is comparable to other similar state-of-the-art unsupervised platforms [36–38]. We anticipate that the adherence levels will be further improved by implementing our approach on mobile devices and making it available on a wider range of operating systems, not just Windows PCs. Furthermore, given that our analysis of inclusion criteria showed that shorter periods of engagement should be sufficient to make a reliable characterization of MTL-related memory, presenting future studies with shorter duration requirements may further increase adherence rates.

Our results show that in line with previous findings on analogous spatial memory tests conducted in clinics and research laboratories, the performance on spatial alternation and double spatial alternation tasks negatively correlates with inter-trial intervals. Importantly, unlike other tests, we could simultaneously sample the whole range (20 seconds—3 hours) of inter-trial intervals. Furthermore, the task difficulty levels could be dynamically adjusted to optimise the spread of scores and avoid both ceiling and floor effects. Namely, the difficulty level could be controlled by introducing more complex rules of spatial alternation or by making the images more similar in image recognition and visuospatial task. Notably, we found strong practice effects on double spatial alternation tasks in older and middle age groups (>50 years old). Previously, improvements in similar spatial memory tasks were identified as potential measures for assessing cognitive decline in MCI patients [20–22].

Unexpectedly, we found that the younger group performed worse than the older group in the Phase 1 AC task. This suggests that even though remote testing may solve frequency and scalability limitations associated with standard neurophysiological assessments, it brings an entirely different set of challenges related to testing in uncontrolled environments. We hypothesise that participants’ ability to solve a given task in real-world environments is often significantly influenced by other factors not directly related to long-term memory, such as cognitive load, external distractions, motivation and compliance. The analysis of questionnaire data further supported this hypothesis. Namely, we found that the younger group tended to randomly press the buttons to proceed with their ongoing activities significantly more frequently than older and middle age groups (Fig 1G). Given such confounds, we anticipate that such unsupervised active testing may be more suitable for older age groups (>50 years old), who may be motivated by their wish to receive an unbiased assessment of their cognitive abilities, which may have clinical relevance. The interest in clinical relevance in older groups was confirmed during the Focus Group discussions.

We also speculate that relative rather than absolute performance measures may be more appropriate in uncontrolled environments. This can be achieved by creating personalised double spatial alternation, image recognition and visuospatial tasks with different degrees of difficulties by adjusting the time intervals between the choices (double spatial alternation task), applying different choice rules or by tuning similarity between the presented images as was done in Phase 1 and Phase 2 image recognition and visuospatial tasks. Such personalised readjustment will impose different cognitive demand required to solve the tasks while keeping other external factors similar. Finally, adding additional validated measures of motivational factors to have an independent way of assessing personal motivation and levels of interference may help to disambiguate memory-related performance from other uncontrolled factors. This can be a few simple questions at the end of each day, possibly combined with passive readings such as time-to-engage, the number of engagements and the reaction time known to be affected by the motivation [39].

In summary, our results show that hAge presents a promising new approach for mass testing MTL-dependent long-term memory under ethological conditions in a remote and unsupervised way. To validate its utility for clinical diagnostics on a large scale, it will have to be tested in clinical populations and implemented on mobile devices instead of desktop PCs. This should dramatically increase its flexibility and adherence level and allow linking the performance outcomes to external factors routinely measured by mobile devices (e.g. sleep, exercise, location etc.). Moreover, the platform will have to be validated against well-established AD biomarkers and correlated with imaging measures of MTL and prefrontal areas.

## Supporting information

S1 Appendix Supplementary tables and figures. (PDF)
